# Evidence of Detrimental Effects of Environmental Contaminants on Growth and Reproductive Physiology of White Sturgeon in Impounded Areas of the Columbia River

**DOI:** 10.1289/ehp.8072

**Published:** 2005-07-11

**Authors:** Grant W. Feist, Molly A.H. Webb, Deke T. Gundersen, Eugene P. Foster, Carl B. Schreck, Alec G. Maule, Martin S. Fitzpatrick

**Affiliations:** 1 Department of Fisheries and Wildlife, Oregon State University, Corvallis, Oregon, USA; 2 Environmental Science Program, Pacific University, Forest Grove, Oregon, USA; 3 Oregon Department of Environmental Quality, Portland, Oregon, USA; 4 Oregon Cooperative Fish and Wildlife Research Unit, Oregon State University, Corvallis, Oregon, USA; 5 Biological Resources Division, U.S. Geological Survey, Corvallis, Oregon, USA; 6 Biological Resources Division, U.S. Geological Survey, Columbia River Research Laboratory, Cook, Washington, USA

**Keywords:** endocrine-disrupting chemicals, growth, PCBs, pesticides, reproductive physiology, sex steroids, white sturgeon

## Abstract

This study sought to determine whether wild white sturgeon from the Columbia River (Oregon) were exhibiting signs of reproductive endocrine disruption. Fish were sampled in the free-flowing portion of the river (where the population is experiencing reproductive success) and from three reservoirs behind hydroelectric dams (where fish have reduced reproductive success). All of the 18 pesticides and almost all of the 28 polychlorinated biphenyls (PCBs) that were analyzed in livers and gonads were detected in at least some of the tissue samples. Metabolites of *p*,*p*′-dichlorodiphenyltrichloroethane (DDT) [*p*,*p*′-dichlorodiphenyldichloroethylene (DDE) and *p*,*p*′-1,1-dichloro-2,2-bis(4-chlorophenyl)ethane (DDD)] were consistently found at relatively high levels in fish. Some males and immature females showed elevated plasma vitellogenin; however, concentrations were not correlated with any of the pesticides or PCBs analyzed. Negative correlations were found between a number of physiologic parameters and tissue burdens of toxicants. Plasma triglycerides and condition factor were negatively correlated with total DDT (DDD + DDE + DDT), total pesticides (all pesticides detected – total DDT), and PCBs. In males, plasma androgens and gonad size were negatively correlated with total DDT, total pesticides, and PCBs. Fish residing in the reservoir behind the oldest dam had the highest contaminant loads and incidence of gonadal abnormalities, and the lowest triglycerides, condition factor, gonad size, and plasma androgens. These data suggest that endocrine-disrupting chemicals may be accumulating behind dams over time. Overall, results of this study indicate that exposure to environmental contaminants may be affecting both growth and reproductive physiology of sturgeon in some areas of the Columbia River.

The lower Columbia River supports one of the most productive white sturgeon (*Acipenser transmontanus*) fisheries in North America (DeVore et al. 1995; McCabe and Tracy 1994). Fish trapped behind the dams of the hydroelectric system, however, have reduced reproductive success compared with animals in the free-flowing portion of the river (Beamesderfer etal. 1995). Reduced reproductive fitness of fish in these impounded sections of the river has been attributed to habitat, flow, and temperature, but environmental toxicants could also be playing a role. The long-lived, late-maturing, and benthic lifestyle of sturgeon may make them particularly susceptible to the actions of persistent bio-accumulating pollutants (DeVore etal. 1995).

The Columbia River receives pollution from a variety of sources that include sewage treatment plants, bleached-kraft pulp mills, aluminum smelters, mining operations, and agricultural and urban runoff. Recently, it has been determined that past operation of the hydroelectric facilities has led to contamination of certain areas of the river with polychlorinated biphenyls (PCBs) (URS Corporation 2002). A wide variety of environmental contaminants have been shown to have adverse effects on reproduction in fishes (Kime 1995; Tyler et al. 1998; Van Der Kraak 1998), and many of these bioaccumulating toxicants have been detected in sediments and fish from the Columbia River [Foster et al. 1999, 2001a, 2001b; U.S. Environmental Protection Agency (EPA) 2002].

This study was designed to examine whether environmental pollutants are having an adverse effect on the reproductive physiology of white sturgeon in the wild and to determine whether fish demonstrate evidence of reproductive endocrine disruption that correlates to specific areas within the river where sturgeon are known to have low reproductive success.

## Materials and Methods

### Fish sampling.

**Figure 1:**
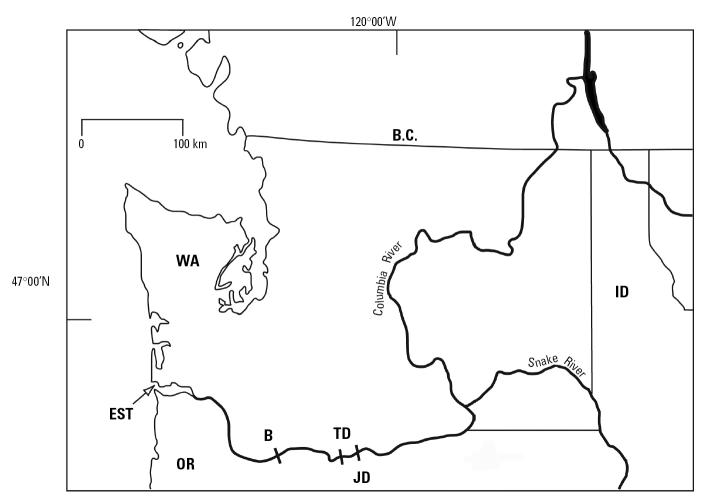
Sample sites for white sturgeon from the Columbia River in the estuary near Astoria, Oregon (EST), and the reservoirs behind Bonneville (B), The Dalles (TD), and John Day (JD) dams. Abbreviations: B.C., British Columbia; ID, Idaho; OR, Oregon; WA, Washington State.

Fish were sampled during the commercial and sport harvest in February through April of 2000 and 2001. Because of state fishing regulations, only fish within a slot limit of 110–137 cm fork length were sampled. This slot limit is set to ensure that mature fish are not removed from the fishery. Fish were sampled from four areas of the Columbia River: the free-flowing portion of the river in the estuary at Astoria, Oregon, and in reservoirs above Bonneville (river mile 191), The Dalles (river mile 216), and John Day (river mile 292) dams (Figure 1). These dams were constructed in 1938, 1960, and 1971, respectively. A total of 174 fish were sampled, representing 42–45 individuals (19–24 males and 21–23 females) for each location. Length and weight were recorded, and condition factor (CF) was determined. Gonads were removed and weighed, and gonadosomatic index (GSI) was determined. Gonads and livers were collected for both histologic and contaminant analysis. Plasma samples were collected for analysis of 17β-estradiol (E_2_), testosterone (T), 11-keto-testosterone (KT), vitellogenin (Vtg), calcium, and triacylglycerides (TAG). In 2001, pectoral fin spines were collected to determine the age of fish.

All animals were treated in accordance with Oregon State University’s Care of Laboratory Animals guidelines (Oregon State University Institutional Animal Care and Use Committe 2005).

### Plasma analyses.

We extracted the steroids T, KT, and E_2_ from plasma following the method of Fitzpatrick etal. (1986). Extraction efficiencies for all steroids were determined by adding tritiated steroids to tubes containing plasma (*n* = 4) during each extraction. This resulted in 12 extraction efficiencies for each steroid. The average extraction efficiencies (ranges) for T, KT, and E_2_ were 92.5 (88.8–94.6), 82.5 (81.6–83.0), and 83.4% (79.8–85.5%), respectively. All steroid assay results were corrected for recovery.

We measured plasma concentrations of T, KT, and E_2_ by radioimmunoassay (RIA) as described by Sower and Schreck (1982) and modified by Feist et al. (1990). All samples were analyzed in duplicate. The lower limit of detection was 1.25 pg/tube for all assays, except KT (3.12 pg/tube). The intra- and interassay coefficients of variation for all assays were < 5 (*n* = 12) and 10% (*n* = 12), respectively. We validated steroid levels determined by RIA by verifying that serial dilutions were parallel to standard curves.

Vtg was measured by enzyme immunoassay following the methodology of Linares-Casenave et al. (1994) and Heppell and Sullivan (1999). Purified white sturgeon Vtg and antibody were a gift from S. Doroshov (University of California–Davis). The lower limit of detection was 3.9 ng/mL, and the assay was validated by verifying that serial dilutions of samples were parallel to the standard curve. The intra- and interassay coefficients of variation were < 5 (*n* = 72) and 10% (*n* = 72), respectively. We determined calcium and TAG plasma content using diagnostic kits from Sigma (587-A and 334-A; St. Louis, MO).

### Histology.

Gonad and liver tissue was stored in 10% phosphate-buffered formalin, embedded in paraffin, sectioned at 7 μm, and stained by hematoxylin and eosin (Luna 1968). Slides were examined under a compound scope (Motic Instruments, Inc., Richmond, B.C., Canada) using 10× to 100× objectives. We scored germ cells for stage of development according to the protocol of Van Eenennaam and Doroshov (1998). Stage 1 (differentiation of testis and ovary) and stage 2 (proliferation of spermatogonia and endogenous growth of the oocyte) fish were immature, whereas stage 3–6 males (onset of meiosis through spermiation) and stage 3–7 females (early vitellogenesis through ovulation) were classified as maturing. Each slide (liver and gonad tissue) was examined completely for presence or absence of gross lesions or other abnormalities, followed by semiquantification of macrophage aggregates (MA) in gonad and liver tissue and of eosinophils and lymphocytes in hepatic tissue in a randomly chosen field of view (10×). We formulated an index for semiquantification for the fish captured in the fisheries: 0, no MA or lymphocytes; 1, 1–25% of the tissue contained MA or lymphocytes; 2, 26–50% of the tissue contained MA or lymphocytes; 3, 51–75% of the tissue contained MA or lymphocytes; 4, 75–100% of the tissue contained MA or lymphocytes.

### Contaminant analysis.

We analyzed a sub-sample of livers (*n* = 97) and gonads (*n* = 98) for 18 chlorinated pesticides and 28 PCB congeners (Appendix 1). This represented 11–17 males and 10–14 females from each sampling location.

Extraction and cleanup procedures of sturgeon tissues were based on the methods described by Price etal. (1986) and Gundersen et al. (1998). Liver and gonad samples were homogenized using a Brinkmann Polytron tissue homogenizer (Brinkmann Instruments, Inc., Westbury, NY), and a portion was removed for measurement of moisture content. Subsamples of tissue homogenates (~ 5 g) were combined with sodium sulfate (~ 50 g) and ground to a fine powder using a mortar and pestle. Dried tissues were Soxhlet extracted (10 hr) with 170 mL of 1:1 petroleum ether/hexane (vol/vol spectral grade; Sigma-Aldrich, St. Louis, MO). Extracts were concentrated to < 15 mL with a rotary evaporator and transferred to tared vials, where the remaining solvent was evaporated to dryness using a warm water bath and a stream of pure nitrogen (N_2_). The amount of lipid in each sample was determined gravimetrically. Lipid extracts were cleaned using 20 g Florisil-packed glass columns (400 × 19 mm), and PCBs and chlorinated pesticides were eluted with 6% ethyl ether/petroleum ether (vol/vol). PCBs and pesticides were fractionated into two eluates using 5 g silica gel-packed glass columns (10.5 × 300 mm). The first fraction [PCBs and *p*,*p*′-dichlorodiphenyldichloroethylene (DDE)] was eluted with hexane. The second fraction (chlorinated pesticides) was eluted with benzene.

We analyzed the cleaned fractions using a Varian CP-3800 gas chromatograph (Varian, Inc., Walnut Creek, CA) equipped with a ^63^Ni electron capture detector, a CP-8200 AutoSampler, a Star Chromatography Workstation (version 5; Varian Inc.), and an SPB-608 fused silica capillary column (30 mm × 0.25 mm × 0.25 μm film thickness; Supelco, Bellefonte, PA). Gas chromatographic parameters used were as follows: carrier gas, helium (1.5 mL/min); makeup gas, nitrogen; detector temperature, 300°C; injector temperature, 290°C; and oven temperature,

Quality assurance measures included the analysis of reagent blanks, duplicates, and matrix spike samples. Percent recoveries of PCB congeners and organochlorine pesticides in matrix spikes were between 90 and 110%; therefore, sample extracts were not corrected for percent recovery. Detection limits for individual PCB congeners and chlorinated pesticides were 0.01 μg/g wet weight. The State of Oregon Environmental Quality Laboratories and Applied Research, Organic Laboratory section (Portland, OR), analyzed two tissue homogenates for chlorinated pesticides (interlaboratory comparison). The relative percent difference of organochlorine pesticide concentrations reported by the two laboratories in the two samples differed by an average of < 17%.

### Aging of fish.

Ages of fish sampled in 2001 were determined by pectoral fin spine analysis following the procedures described by Beamesderfer et al. (1989). Two independent determinations were conducted at the Oregon Department of Fish and Wildlife (Clackamas, OR) and at University of California–Davis (Davis, CA). Of the fish, 27% had identical age assignments by the different readers, 45% were aged within 1 year, 22% within 2 years, 2% within 3 years, and 4% > 5 years. We averaged ages of fish that were not in agreement between the two determinations.

### Western blot analysis.

Hepatic microsomes were prepared by differential centrifugation according to Carpenter et al. (1990) and stored at −80°C until use. Briefly, livers were minced in ice-cold buffer (0.1 M Tris-acetate, pH 7.4; 0.1 M KCl; 1 mM EDTA; 20 μM butylated hydroxytoluene; and 1 mM phenyl-methylsulfonylfluoride) and homogenized in 4 volumes of the same buffer. The homogenate was centrifuged at 10,000 × *g* for 30 min, and the resulting supernatant was centrifuged at 100,000 × *g* for 90 min. The microsomal pellet was resuspended in buffer (0.1 M phosphate buffer, pH 7.25; 20% glycerol; and 1 mM EDTA). Microsomes were stored at −80°C until use.

We measured the putative white sturgeon hepatic cytochrome P450 3A (CYP3A) enzyme in microsomes by Western blotting using a polyclonal antibody generated against rainbow trout LMC5 (3A27). Microsomal CYP3A protein was measured using Western immunoblot techniques according to Towbin et al. (1979) with modifications. Briefly, sodium dodecyl sulfate polyacrylamide gel electrophoresis (SDS-PAGE) was performed using 8% polyacrylamide precast minigels. We prepared membranes according to the manufacturers recommendations, and proteins were transferred to membranes followed by incubation with rabbit anti-trout antibody (a generous gift from D. Buhler). Membranes were rinsed with phosphate-buffered saline–Tween and incubated with horseradish peroxidase–conjugated secondary antibodies (anti-rabbit) for detection of oxidized luminol (Amersham Biosciences, Piscataway, NJ). The chemiluminescent signal was captured on film (Hyperfilm ECL, Amersham Biosciences), and films were scanned for quantification.

### Statistics.

We conducted all mean comparisons between physiologic parameters, tissue contaminant load, river location, and sex of fish using a one-way analysis of variance (ANOVA) with a Bonferroni procedure. All correlations between tissue contaminant load and physiologic parameters were conducted using reciprocal-Y regression. We performed all analyses using Statview software (Abacus Concepts, Inc., Berkeley, CA), and the accepted level of significance for all tests was *p* < 0.05.

## Results

**Figure 2:**
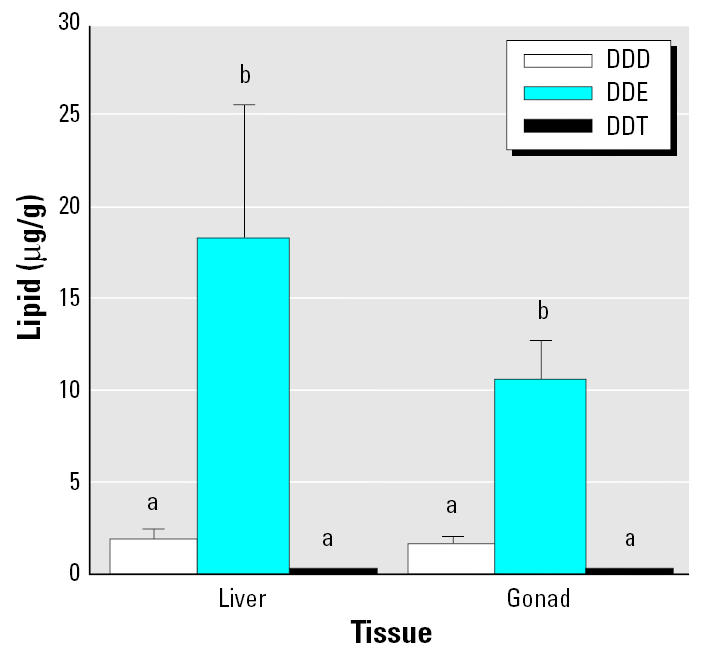
Mean concentrations (± SE) of DDT and its metabolites in livers (*n* = 97) and gonads (*n* = 98) of white sturgeon from all sample areas combined. Means with different letters indicate a significant difference within a tissue (ANOVA, *p* < 0.05).

All 18 of the chlorinated pesticides examined in tissues from wild fish were detected in at least some of the samples (Table 1). We consistently found relatively high levels of metabolites of *p*,*p*′-dichlorodiphenyl-trichloroethane (DDT) [DDE and *p*,*p*′-1,1-dichloro-2,2-bis(4-chlorophenyl)ethane (DDD)] in fish. Concentrations of DDE were always greater than those of DDD and DDT in both livers and gonads (Figure 2). We found no differences in toxicant levels between tissues. Of the 28 PCB congeners examined, 26 were detected in at least some of the samples (Table 2).

**Figure 3:**
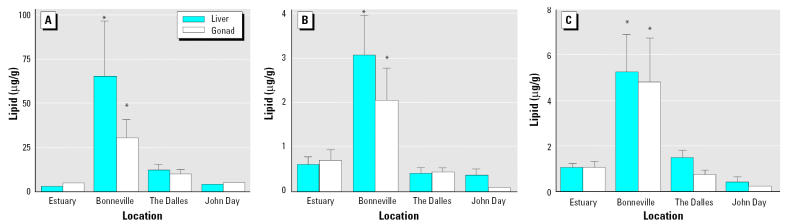
Concentrations (mean ± SE) of total DDT (*A*; DDD + DDE + DDT), total pesticides (*B*; all pesticides detected – total DDT), and PCBs (*C*; total of all detected) in livers and gonads of white sturgeon from four locations on the Columbia River. Each bar represents a sample size of 22–28. *Statistically different from other locations (ANOVA, *p* < 0.05).

**Figure 4:**
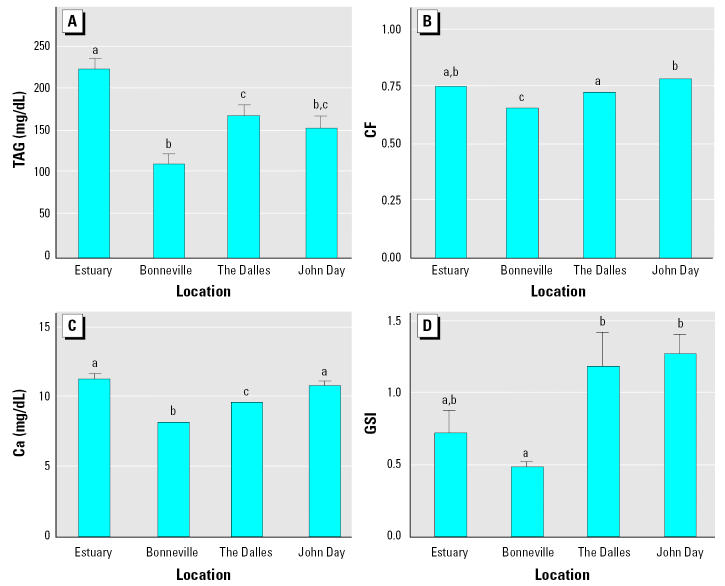
Mean plasma concentrations (± SE) of TAG (*A*), CF (*B*), calcium (*C*), and GSI (*D*) in white sturgeon from four locations on the Columbia River. Each bar represents a sample size of 42–45. Means with different letters indicate a significant difference between locations (ANOVA, *p* < 0.05).

Total DDT (DDD + DDE + DDT), total pesticides (all pesticides detected – total DDT), and PCBs (total of all detected) were significantly higher in livers and gonads of fish from Bonneville Reservoir compared with other locations (Figure 3). Fish from the Bonneville Reservoir had significantly lower TAG plasma concentrations and GSI than two of the other locations (Figure 4). Fish from Bonneville also had significantly lower calcium plasma concentrations and CF compared with all other locations.

We found a negative correlation between plasma TAG and total DDT, pesticides, and PCBs in livers (Table 3). To varying degrees, this was also true for TAG compared with contaminants in gonads and for CF compared with contaminants in livers and gonads. Although we observed significant relationships, *r*^2^ values indicated that a large amount of variation was present within the data.

**Figure 5:**
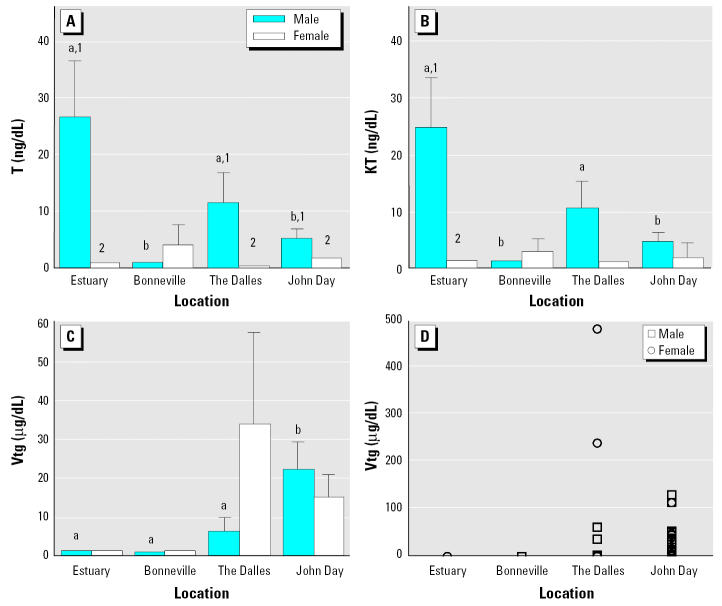
Mean plasma concentrations (± SE) of T (*A*), KT (*B*), and Vtg (*C*) and individual Vtg concentrations (*D*) in male and immature female white sturgeon from four locations on the Columbia River. Each bar represents a sample size of 19–24 (*A–C*). Means with different letters or numbers indicate a significant difference between locations or between sexes within a location, respectively (ANOVA, *p* < 0.05).

Plasma concentrations of T were higher in males than in females at all sample locations except Bonneville (Figure 5). Males from the estuary had significantly higher levels of KT than did females, but this was not observed at other locations. Males from the estuary had significantly higher plasma T and KT than did males in the Bonneville and John Day reservoirs. Plasma concentrations of E_2_ were very low in all fish examined (Table 4). We observed no differences between either sex or location.

Plasma Vtg was at or very near the detection limit of the assay for all fish sampled in the estuary and Bonneville (Figure 5). Some males and immature females from The Dalles and John Day reservoirs had detectable levels of Vtg. Males from John Day had significantly higher concentrations of Vtg than did fish from all other locations. Females from The Dalles had concentrations of Vtg that were nearly significant compared with females from the estuary (*p* = 0.060) as well as compared with females from Bonneville (*p* = 0.058). There was no correlation between plasma Vtg and any of the pesticides or PCBs that were monitored.

Gonadal histology revealed a total of 82 females, 73 males, and 3 hermaphrodites from the 2 years of sampling. Sixteen gonad samples contained only adipose tissue and no gonial cells. Of the females, 81 were immature (all stage 2 except for 3 stage 1 females), and 1 was a maturing female (stage 3; early vitellogenesis). Of the males, 66 were immature (all stage 2), 1 was in stage 3 of gonadal development (onset of meiosis), and 6 were in stage 5 of development (spermiation). No maturing fish were captured in Bonneville Reservoir. All of the maturing males had significantly higher levels of plasma androgens (T, 92.2 ± 20.9; KT, 84.0 ± 16.4 ng/mL) than did immature males (T, 5.1 ± 1.1; KT, 4.3 ± 1.0 ng/mL). All 3 of the hermaphroditic fish had predominately female ovotestes. Two of the 3 fish were captured in Bonneville Reservoir, and the other was from the estuary. Several fish showed irregular ovarian plasma membranes and intrusion of muscle into the ovary. MAs were found in both female and male gonadal tissue and were most often found to contain melanin.

Liver histology revealed a high incidence of MA and lymphocytes. However, no pattern was discernible with regard to contaminant level. We found a very high incidence of MA and/or lymphocytes in liver samples from 11 fish; of these, 7 were from the Bonneville Reservoir, 2 were from the estuary, and 1 each were from The Dalles and John Day reservoirs.

**Figure 6:**
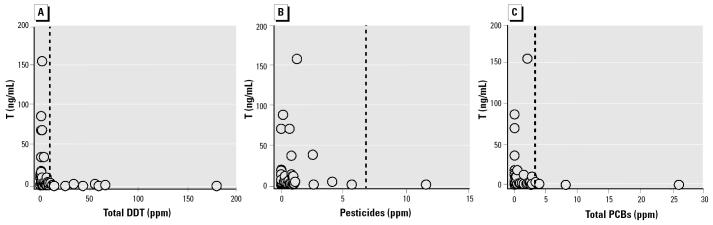
Individual plasma T versus total DDT (*A*), total pesticides (*B*), or total PCB (*C*) concentrations in livers of male white sturgeon. Reciprocal-Y regression: *p* < 0.001 and *r*^2^ = 0.79 for DDT, *p* < 0.001 and *r*^2^ = 0.56 for pesticides, and *p* < 0.001 and *r*^2^ = 0.80 for PCBs. All males with toxicant levels higher than those denoted by the vertical dashed line have < 4 ng/mL T.

**Figure 7:**
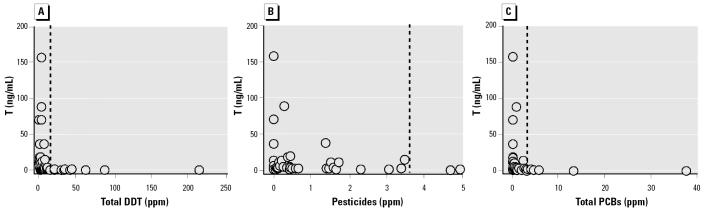
Individual plasma T versus total DDT (*A*), total pesticides (*B*), or total PCB (*C*) concentrations in gonads of male white sturgeon. Reciprocal-Y regression: *p* < 0.001 and *r*^2^ = 0.85 for DDT, *p* < 0.001 and *r*^2^ = 0.31 for pesticides, and *p* < 0.001 and *r*^2^ = 0.82 for PCBs. All males with toxicant levels higher than those denoted by the vertical dashed line have < 4 ng/mL T.

We found a negative correlation between plasma T and total DDT, pesticides, and PCBs in livers of male white sturgeon (Figure 6). We also observed these relationships for contaminants in gonads (Figure 7). To varying degrees, this was also true for plasma KT and GSI compared with contaminants in gonads and livers (Table 5).

Spermatogonia proliferation (stage 2) in white sturgeon is associated with increased circulating androgen concentrations regardless of age or size (Feist et al. 2004). In immature wild white sturgeon, T concentrations > 4 ng/mL may be used to differentiate stage 2 males from stage 1 males and immature females (Webb et al. 2002). All 66 immature males in our study were in stage 2 of gonadal development, yet 47 (71.2%) had plasma T concentrations that were < 4 ng/mL. Of the 48 stage 2 males that were analyzed for toxicants, 31 had levels of T < 4 ng/mL. In addition, no males with liver contaminant levels > 9.5 ppm (total DDT), > 5.6 ppm (total pesticides), or > 2.8 ppm (PCBs) had plasma T concentrations > 4 ng/mL (Figure 6). Where this was observed, concentrations of toxicants in gonads were 11.6, 3.7, and 2.5 ppm, for total DDT, total pesticides, and PCBs, respectively (Figure 7).

Age determination of fish by pectoral fin spine analysis in 2001 revealed that sturgeon from Bonneville (18.3 ± 1.0 years; range, 14–27) and John Day (17.4 ± 0.4 years; range, 14–20) were significantly older than those sampled in The Dalles (14.8 ± 0.5 years; range, 10–19). Bonneville fish were also significantly older than estuary fish (14.6 ± 1.0 years; range, 10–17).

**Figure 8:**
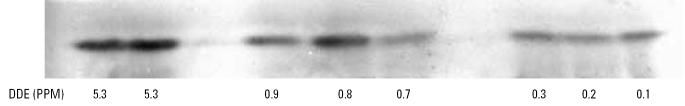
Western blot of CYP3A protein in individual livers of male white sturgeon with varying levels of liver DDE.

To investigate the possibility that DDE reduces plasma androgens by increasing steroid metabolism and excretion via up-regulation of liver cytochrome P450 isozymes, we conducted a preliminary and purely qualitative Western blot analysis to measure the putative CYP3A in microsomes. In trout this enzyme is responsible for hydroxylating steroids as a first step for metabolism and excretion (Lee et al. 2001). A Western blot for this isozyme is shown in Figure 8. Male sturgeon with higher liver content of DDE showed increased immunoreactivity for CYP3A.

## Discussion

The life history of white sturgeon may make them particularly susceptible to the actions of persistent bioaccumulating pollutants. These fish are bottom dwellers and feed on benthic prey items that are closely associated with sediments containing hydrophobic pollutants. Sturgeon can live for > 100 years, and females mature between 16 and 35 years of age (DeVore et al. 1995). Thus, toxicants may accumulate and have deleterious effects over a long period of time before the fish reach a stage when they are able to reproduce. A recent study in the Columbia River found that sturgeon contained the highest body burdens of contaminants out of 12 species of fish examined (U.S. EPA 2002). Levels of toxicants seen in the present study were comparable with those found by the U.S. EPA and also comparable with levels previously reported by our laboratory (Foster et al. 2001a, 2001b).

Fish trapped behind the oldest of the dams examined (Bonneville) had the highest contaminant loads and the lowest CF, gonad size, and plasma androgens and triglycerides. These fish also had the highest incidence of gonadal abnormalities. This suggests that endocrine-disrupting chemicals (EDCs) may be accumulating behind dams over time.

It has recently been determined that past operation of the dam at Bonneville has resulted in areas within the reservoir that have very high levels of PCBs (URS Corporation 2002). In our study, Bonneville fish were older than fish from two of the other sampling locations. Fish from this reservoir also grow slower, and females mature at a later age than other locations (Beamesderfer et al. 1995). Thus, these fish may be exposed to higher levels of contaminants and for longer periods of time than comparably sized fish from other areas of the river. Food availability may be the main cause for reduced growth in Bonneville fish, but effects of toxicants cannot be ruled out. The negative correlations found between plasma triglycerides and CF with tissue burdens of pesticides and PCBs add strength to this possibility.

Our laboratory has previously documented a negative correlation between plasma androgens and tissue content of *p*,*p*′-DDE for Columbia River sturgeon (Foster et al. 2001b). In the present study, we observed negative correlations between both plasma androgens and GSI of males compared with total DDT, total pesticides, and PCBs. Our sample size for this study was much greater than our previous research, which may explain why these relationships were not seen in the earlier study. *p*,*p*′-DDE has also been shown to have demasculinizing effects in the guppy (*Poecilia reticulata*) (Baatrup and Junge 2001; Bayley et al. 2002). Our data also suggest that DDT and its metabolites may reach threshold levels in liver and gonad above which the fish are incapable of elevating plasma T concentrations. This may result in the inability of males with high body burdens of contaminants to attain sexual maturity.

We have preliminary evidence that the mechanism of action of plasma androgen reduction by *p*,*p*′-DDE, or possibly by other pesticides or PCBs, is by increasing steroid metabolism through up-regulation of CYP3A. DDE has been shown to induce this isozyme and increase metabolism of T in mice (Dai et al. 2001). Rainbow trout (*Oncorhynchus mykiss*) injected with DDE, however, showed a decrease in CYP3A-dependent 6β-hydroxylation of T (Machala et al. 1998). The dose used for the rainbow trout study was much higher (50 mg/kg) than levels seen in wild fish in our study and may not have simulated the effects of chronic exposure to lower concentrations of DDE.

Our finding that plasma androgens were higher in males than females (except in the Bonneville Reservoir) has been previously documented by our laboratory (Foster et al. 2001a, 2001b). We have used differences in plasma steroids between males and females to develop a model for sexing both immature and maturing wild white sturgeon and for determining sex of cultured fish at an early age (Feist et al. 2004; Webb etal. 2002).

Although banned for use in the United States in 1973, DDT and its metabolites are still being detected in sturgeon at relatively high levels. This indicates that this compound is extremely persistent in the environment. Tissue burdens were always DDE > DDD and DDT, indicating that aerobic degradation of DDT (yielding primarily DDE) is the main metabolic pathway as opposed to anaerobic degradation (yielding primarily DDD) (Spencer etal. 1996). This suggests that the most likely source of DDT metabolites is from agricultural runoff of the parent compound as opposed to anaerobic degradation of DDT in sediments.

The type and source of xenoestrogen(s) responsible for elevating plasma Vtg in males and immature females from The Dalles and John Day reservoirs remains uncertain. None of the pesticides or PCBs monitored in this study was correlated with plasma Vtg. Fish exposed in our laboratory to the pesticides (permethrin and pyriproxyfen) or herbicides (atrazine and simazine) that are currently being used in agricultural practices in the Columbia basin did not show increases in plasma Vtg (data not shown). Caged sturgeon, in areas of the river where some wild fish had elevated Vtg, also did not show an increase in this protein (data not shown). This suggests that wild fish either are being exposed to potential EDCs for longer periods of time or are bioaccumulating them through ingestion of prey.

Other candidates for induction of Vtg include the alkylphenols, which have been shown to be weakly estrogenic in fish (Jobling etal. 1996; White etal. 1994). Fish exposed to octylphenol and nonylphenol in our laboratory experienced increased plasma Vtg (data not shown), but we are unable to find a likely source for alkylphenolic compounds in The Dalles and John Day reservoirs. There are many sources of alkylphenols in the estuary and Bonneville Reservoir, yet we found no elevated Vtg in wild sturgeon sampled in this area of the river. The cause of elevated Vtg in wild fish is most likely due to other EDCs or metabolites of toxicants not yet identified, or combinations of compounds.

The overall results of this study indicate that exposure to environmental contaminants may be affecting both growth and reproductive physiology of sturgeon in some areas of the Columbia River. Questions remain, however, as to what effects these contaminants have on the ability of sturgeon to successfully reproduce. It is unknown if lowered energy reserves, GSI, and androgens, and elevated Vtg actually inhibit or decrease the ability of sturgeon to mature and spawn. Because of the slot-size limit (fish that are 110–137 cm in fork length), most wild fish sampled in this study were immature. Larger sturgeon that have reached a sufficient size and age to mature must be examined to determine possible deleterious effects of contaminants on reproduction. Different year classes of sturgeon also need to be investigated to determine if toxicants are bioaccumulating as the fish age. Finally, prey items need to be examined for the presence of EDCs to determine if sturgeon are acquiring these compounds from their diet or other sources.

The poor reproductive success of sturgeon in impounded areas of the Columbia River is most likely due to a wide variety of stressors, including food availability, poor spawning habitat, and changes in flow and temperature. Exposure to environmental contaminants may be an additional stressor that is contributing to this reduced reproductive fitness.

## Figures and Tables

**Table 1:** Concentration (mean ± SE) of chlorinated pesticides in livers (*n* = 97) and gonads (*n* = 98) of white sturgeon from the Columbia River.

	Liver		Gonad	
Pesticide	D	Lipid (μg/g)	D	Lipid (μg/g)
Aldrin	2	0.002 ± 0.002	5	0.011 ± 0.006
α-BHC	19	0.039 ± 0.009	26	0.023 ± 0.005
β-BHC	14	0.115 ± 0.046	11	0.023 ± 0.005
γ -BHC	8	0.024 ± 0.011	21	0.047 ± 0.014
δ -BHC	9	0.019 ± 0.007	15	0.154 ± 0.127
*p*,*p*′-DDD	86	1.863 ± 0.544	93	1.619 ± 0.400
*p*,*p*′-DDE	97	18.40 ± 7.313	98	10.60 ± 2.086
*p*,*p*′-DDT	28	0.274 ± 0.103	41	0.259 ± 0.073
Dieldrin	16	0.134 ± 0.045	15	0.031 ± 0.009
Endrin	10	0.114 ± 0.060	11	0.022 ± 0.007
Endrin aldehyde	16	0.108 ± 0.062	13	0.064 ± 0.032
Endrine ketone	8	0.038 ± 0.165	2	0.010 ± 0.007
Endosulfan I	34	0.161 ± 0.044	45	0.133 ± 0.025
Endosulfan II	9	0.108 ± 0.051	14	0.087 ± 0.047
Endosulfan sulfate	3	0.005 ± 0.003	8	0.008 ± 0.003
Heptachlor	8	0.018 ± 0.008	13	0.037 ± 0.019
Heptachlor epoxide	15	0.081 ± 0.031	25	0.074 ± 0.024
*p*,*p*′-Methoxychlor	14	0.112 ± 0.044	5	0.027 ± 0.017

Abbreviations: BHC, benzene hexachloride; D, number of detections.

**Table 2:** Concentration (mean ± SE) of PCBs in livers (*n* = 97) and gonads (*n* = 98) of white sturgeon from the Columbia River.

	Liver		Gonad	
Pesticide (IUPAC no.)	D	Lipid (μg/g)	D	Lipid (μg/g)
28	3	0.020 ± 0.011	0	
44	6	0.055 ± 0.042	4	0.004 ± 0.002
52	3	0.038 ± 0.024	3	0.024 ± 0.105
60	19	0.125 ± 0.033	11	0.163 ± 0.129
66	8	0.131 ± 0.066	2	0.025 ± 0.020
74	2	0.008 ± 0.006	4	0.037 ± 0.022
87	1	0.006 ± 0.006	2	0.008 ± 0.006
99	12	0.101 ± 0.036	12	0.077 ± 0.041
101	28	0.238 ± 0.088	24	0.217 ± 0.131
105	14	0.135 ± 0.051	9	0.033 ± 0.016
110/77	12	0.060 ± 0.019	17	0.128 ± 0.050
118	9	0.054 ± 0.020	10	0.152 ± 0.085
126	6	0.035 ± 0.016	5	0.024 ± 0.018
128	1	0.007 ± 0.007	6	0.043 ± 0.031
138	28	0.258 ± 0.071	28	0.233 ± 0.072
151	4	0.025 ± 0.015	7	0.032 ± 0.014
153	18	0.264 ± 0.101	20	0.157 ± 0.062
156	6	0.035 ± 0.018	7	0.013 ± 0.006
169	2	0.007 ± 0.005	0	
170	3	0.006 ± 0.003	3	0.003 ± 0.001
180	3	0.030 ± 0.026	3	0.001 ± 0.001
183	9	0.042 ± 0.015	13	0.029 ± 0.010
187	20	0.163 ± 0.047	21	0.113 ± 0.032
194	4	0.018 ± 0.009	1	0.001 ± 0.001
199	10	0.022 ± 0.007	10	0.065 ± 0.030
203/170	10	0.043 ± 0.017	10	0.016 ± 0.008

Abbreviations: D, number of detections; IUPAC, International Union of Pure and Applied Chemistry.

**Table 3:** Regression analyses of TAG and CF versus various contaminants in livers and gonads of Columbia River white sturgeon.

	Liver				Gonad			
	TAG		CF		TAG		CF	
Contaminant	*r*^2^	*p*-Value	*r*^2^	*p*-Value	*r*^2^	*p*-Value	*r*^2^	*p*-Value
Total DDT	0.60	< 0.001	0.08	< 0.005	0.20	< 0.001	0.11	< 0.001
Total pesticides	0.48	< 0.001	0.15	< 0.001	0.04	< 0.050	0.18	< 0.001
Total PCBs	0.60	< 0.001	0.11	< 0.002	0.10	< 0.002	0.07	< 0.008

**Table 4:** Concentration (mean ± SE) of plasma E_2_ (ng/mL) in male (*n* = 19–24) and female (*n* = 21–23) white sturgeon at four locations from the Columbia River.

	Estuary	Bonneville	The Dalles	John Day
Female	0.09 ± 0.02	0.11 ± 0.03	0.13 ± 0.02	0.28 ± 0.05
Male	0.16 ± 0.03	0.07 ± 0.01	0.14 ± 0.03	0.38 ± 0.10

**Table 5:** Regression analyses of KT and GSI versus various contaminants in livers and gonads of male Columbia River white sturgeon.

	Liver				Gonad			
	KT		GSI		KT		GSI	
Contaminant	*r*^2^	*p*-Value	*r*^2^	*p*-Value	*r*^2^	*p*-Value	*r*^2^	*p*-Value
Total DDT	0.08	< 0.050	0.24	< 0.001	0.11	< 0.020	0.21	< 0.001
Total pesticides	NS	NS	0.15	< 0.006	NS	NS	0.22	< 0.001
Total PCBs	0.16	< 0.004	NS	NS	NS	NS	0.10	< 0.030

NS, not significant.

**Appendix 1:** Chlorinated pesticides and PCBs measured in Columbia River white sturgeon livers and gonads.

Chlorinated pesticide	PCB (IUPAC no.)
Aldrin	2,2′,5-Trichlorobiphenyl (18)
α-BHC	2,4,4′-Trichlorobiphenyl (28)
β-BHC	2,2′,3,5′-Tetrachlorobiphenyl (44)
γ-BHC	2,2′,5,5′-Tetrachlorobiphenyl (52)
δ-BHC	2,3,4,4′-Tetrachlorobiphenyl (60)
*p*,*p*′-DDD	2,3′,4,4′-Tetrachlorobiphenyl (66)
*p*,*p*′-DDE	2,4,4′,5-Tetrachlorobiphenyl (74)
*p*,*p*′-DDT	3,3′,4,4′-Tetrachlorobiphenyl (77)
Dieldrin	2,2′,3,4,5′-Pentachlorobiphenyl (87)
Endrin	2,2′,4,4′,5-Pentachlorobiphenyl (99)
Endrin aldehyde	2,2′,4,5,5′-Pentachlorobiphenyl (101)
Endrine ketone	2,3,3′,4,4′-Pentachlorobiphenyl (105)
Endosulfan I	2,3,3′,4′,6-Pentachlorobiphenyl (110)
Endosulfan II	2,3′,4,4′,5-Pentachlorobiphenyl (118)
Endosulfan sulfate	3,3′,4,4′,5-Pentachlorobiphenyl (126)
Heptachlor	2,2′,3,3′,4,4′-Hexachlorobiphenyl (128)
Heptachlor epoxide	2,2′,3,4,4′,5′-Hexachlorobiphenyl (138)
*p*,*p*′-Methoxychlor	2,2′,3,5,5′,6-Hexachlorobiphenyl (151)
	2,2′,4,4′,5,5′-Hexachlorobiphenyl (153)
	2,3,3′,4,4′,5-Hexachlorobiphenyl (156)
	3,3′,4,4′,5,5′-Hexachlorobiphenyl (169)
	2,2′,3,3′,4,4′,5-Heptachlorobiphenyl (170)
	2,2′,3,4,4′,5,5′-Heptachlorobiphenyl (180)
	2,2′,3,4,4′,5′,6-Heptachlorobiphenyl (183)
	2,2′,3,4′,5,5′,6-Heptachlorobiphenyl (187)
	2,2′,3,3′,4,4′,5,5′-Octachlorobiphenyl (194)
	2,2′,3,3′,4,5,5′,6′-Octachlorobiphenyl (199)
	2,2′,3,4,4′,5,5′,6-Octachlorobiphenyl (203)

Abbreviations: BHC, benzene hexachloride; IUPAC, International Union of Pure and Applied Chemistry.

## References

[b1-ehp0113-001675] Baatrup E, Junge M (2001). Antiandrogenic pesticides disrupt sexual characteristics in the adult male guppy (*Poecilia reticulata*). Environ Health Perspect.

[b2-ehp0113-001675] Bayley M, Junge M, Baatrup E (2002). Exposure of juvenile guppies to three antiandrogens causes demasculinization and a reduced sperm count in adult males. Aquat Toxicol.

[b3-ehp0113-001675] Beamesderfer RCP Elliot JC Foster CA 1989. Report A. In: Status and Habitat Requirements of White Sturgeon Populations in the Columbia River Downstream from McNary Dam (Nigro AA, ed). Portland, OR:Bonneville Power Administration, 5–52.

[b4-ehp0113-001675] Beamesderfer RCP, Rien TA, Nigro AA (1995). Dynamics and potential production of white sturgeon populations in three Columbia River reservoirs. Trans Am Fish Soc.

[b5-ehp0113-001675] Carpenter HM, Fredrickson LS, Williams DE, Buhler DR, Curtis LR (1990). The effect of thermal acclimation on the activity of aryl-hydrocarbon hydroxylase in rainbow trout (*Oncorhynchus mykiss*). Comp Biochem Physiol.

[b6-ehp0113-001675] Dai D, Cao Y, Falls G, Levi PE, Hodgson E, Rose RL (2001). Modulation of mouse P450 isoforms CYP1A2, CYP2B10, CYP2E1, and CYP3A by the environmental chemicals mirex, 2,2-bis(p-chlorophenyl)-1,1-dichloroethylene, vinclozolin, and flutamide. Pestic Biochem Physiol.

[b7-ehp0113-001675] DeVore JD, James BW, Tracy CA, Hale DA (1995). Dynamics and potential production of white sturgeon in the unimpounded Lower Columbia River. Trans Am Fish Soc.

[b8-ehp0113-001675] Feist G, Schreck CB, Fitzpatrick MS, Redding JM (1990). Whole body sex steroid concentrations and gonadal histology in coho salmon during sexual differentiation. Gen Comp Endocrinol.

[b9-ehp0113-001675] Feist GW, Van Eenennaam JP, Doroshov SI, Schreck CB, Schneider RP, Fitzpatrick MS (2004). Early identification of sex in cultured white sturgeon, *Acipenser transmontanus*, using plasma steroid levels. Aquaculture.

[b10-ehp0113-001675] Fitzpatrick MS, Van Der Kraak G, Schreck CB (1986). Profiles of plasma sex steroids and gonadotropin in coho salmon, *Oncorhynchus kisutch*, during final maturation. Gen Comp Endocrinol.

[b11-ehp0113-001675] Foster EP, Drake D, Farlow R (1999). Polychlorinated dibenzo-*p*-dioxin and polychlorinated dibenzofuran congener profiles in fish, crayfish, and sediment collected near a wood treating facility and a bleached kraft pulp mill. Bull Environ Contam Toxicol.

[b12-ehp0113-001675] Foster EP, Fitzpatrick MS, Feist GW, Schreck CB, Yates J (2001a). Gonad organochlorine concentrations and plasma steroid levels in white sturgeon (*Acipenser transmontanus*) from the Columbia River. Bull Environ Contam Toxicol.

[b13-ehp0113-001675] Foster EP, Fitzpatrick MS, Feist GW, Schreck CB, Yates J, Spitsbergen JM (2001b). Plasma androgen correlation, EROD induction, reduced condition factor, and the occurrence of organochlorine pollutants in reproductively immature white sturgeon (*Acipenser transmontanus*) from the Columbia River, USA. Arch Environ Contam Toxicol.

[b14-ehp0113-001675] Gundersen DG, Krahling MD, Donosky JJ, Cable RG, Mims SD (1998). Polychlorinated biphenyls and chlordane in the gonads of paddlefish, *Polyodon spathula*, from the Ohio River. Bull Environ Contam Toxicol.

[b15-ehp0113-001675] Heppell SA, Sullivan CV (1999). Gag (*Mycteroperca microlepis*) vitellogenin: purification, characterization, and use for enzyme-linked immunosorbant assay (ELISA) of female maturity in three species of grouper. Fish Physiol Biochem.

[b16-ehp0113-001675] Jobling S, Sheahan D, Osborne JA, Matthiessen P, Sumpter JP (1996). Inhibition of testicular growth in rainbow trout (*Oncorhynchus mykiss*) exposed to estrogenic alkylphenolic chemicals. Environ Toxicol Chem.

[b17-ehp0113-001675] Kime DE (1995). The effects of pollution on reproduction in fish. Rev Fish Biol Fish.

[b18-ehp0113-001675] Lee S, Hedstrom OR, Fischer K, Wang-Buhler JL, Sen A, Cok I, etal (2001). Immunohistochemical localization and differential expression of cytochrome P450 3A27 in the gastrointestinal tract of rainbow trout. Toxicol Appl Pharmacol.

[b19-ehp0113-001675] Linares-Casenave J Kroll KJ Van Eenennaam JP Doroshov SI 1994. Development and application of an enzyme linked immunosorbent assay (ELISA) for the detection of plasma vitellogenin in white sturgeon (*Acipenser transmontanus*). In: High Performance Fish, Proceedings of an International Fish Physiology Symposium, July 1994, Vancouver, British Columbia, Canada. Vancouver:Fish Physiology Association, 165–169.

[b20-ehp0113-001675] Luna LG 1968. Manual of Histological Staining Methods of the Armed Forces Institute of Pathology. 3rd ed. New York:McGraw-Hill.

[b21-ehp0113-001675] Machala M, Drabek P, Neca J, Kolaova J, Svobodova Z (1998). Biochemical markers for differentiation of exposures to nonplanar polychlorinated biphenyls, organochlorine pesticides, or 2,3,7,8-tetrachlorodibenzo-*p*-dioxin in trout liver. Ecotoxicol Environ Saf.

[b22-ehp0113-001675] McCabe GT, Tracy CA (1994). Spawning and early life history of white sturgeon, *Acipenser transmontanus*, in the lower Columbia River. Fish Bull.

[b23-ehp0113-001675] Oregon State University Institutional Animal Care and Use Committee 2005. Animal Care and Use Form Proposal. Corvallis, OR:Orgegon State University. Available: http://oregonstate.edu/research/osprc/rc/animal/use.html [accessed 1 October 2005].

[b24-ehp0113-001675] Price HA, Welch RL, Scheel RH, Warren LA (1986). Modified multiresidue method for chlordane, toxaphene and polychlorinated biphenyls in fish. Bull Environ Contam Toxicol.

[b25-ehp0113-001675] Sower SA, Schreck CB (1982). Steroid and thyroid hormones during sexual maturation of coho salmon (*Oncorhynchus kisutch*) in saltwater or freshwater. Gen Comp Endocrinol.

[b26-ehp0113-001675] Spencer WF, Singh G, Taylor CD, LeMert RA, Cliath MM, Farmer WJ (1996). DDT persistence and volatility as affected by management practices after 23 years. J Environ Qual.

[b27-ehp0113-001675] Towbin H, Staehlin T, Gordon J (1979). Electrophoretic transfer of proteins from polyacrylamide gels to nitrocellulose sheets: procedure and some applications. Proc Natl Acad Sci USA.

[b28-ehp0113-001675] Tyler CR, Jobling S, Sumpter JP (1998). Endocrine disruption in wildlife: a critical review of the evidence. Crit Rev Toxicol.

[b29-ehp0113-001675] URS 2002. In Water Investigation Report: Bradford Island Landfill, Cascade Locks, Oregon. Portland, OR:URS Corporation.

[b30-ehp0113-001675] U.S. EPA 2002. Columbia River Basin Fish Contaminant Survey, 1996–2002. Seattle, WA:U.S. Environmental Protection Agency, Region 10.

[b31-ehp0113-001675] Van Der Kraak G (1998). Observations of endocrine effects in wildlife with evidence of their causation. Pure Appl Chem.

[b32-ehp0113-001675] Van Eenennaam JP, Doroshov SI (1998). Effects of age and body size on gonadal development of Atlantic sturgeon. J Fish Biol.

[b33-ehp0113-001675] Webb MAH, Feist GW, Foster EP, Schreck CB, Fitzpatrick MS (2002). Potential classification of sex and stage of gonadal maturity of wild white sturgeon using blood plasma indicators. Trans Am Fish Soc.

[b34-ehp0113-001675] White R, Jobling S, Hoare SA, Sumpter JP, Parker MG (1994). Environmentally persistent alkylphenolic compounds are estrogenic. Endocrinology.

